# Innate immune receptors are differentially expressed in mice during experimental *Schistosoma mansoni* early infection

**DOI:** 10.1590/0074-02760240013

**Published:** 2024-06-17

**Authors:** Janete Cunha Lima, Ramayana Morais de Medeiros Brito, Luanderson Cardoso Pereira, Nathalie de Sena Pereira, Manuela Sales Lima Nascimento, Alan Lane de Melo, Paulo Marcos Matta Guedes

**Affiliations:** 1 Universidade Federal do Rio Grande do Norte, Programa de Pós-Graduação em Biologia Parasitária, Natal, RN, Brasil; 2 Universidade Federal de Minas Gerais, Departamento de Parasitologia, Belo Horizonte, MG, Brasil; 3 Universidade Federal do Rio Grande do Norte, Departamento de Microbiologia e Parasitologia, Natal, RN, Brasil

**Keywords:** Schistosomiasis, innate immune receptors, inflammasome, mouse, cytokines

## Abstract

**BACKGROUND:**

The impact of *Schistosoma mansoni* infection over the immune response and the mechanisms involved in pathogenesis are not yet completely understood.

**OBJECTIVES:**

This study aimed to evaluate the expression of innate immune receptors in three distinct mouse lineages (BALB/c, C57BL/6 and Swiss) during experimental *S. mansoni* infection with LE strain*.*

**METHODS:**

The parasite burden, intestinal tissue oogram and presence of hepatic granulomas were evaluated at 7- and 12-weeks post infection (wpi). The mRNA expression for innate Toll-like receptors, Nod-like receptors, their adaptor molecules, and cytokines were determined at 2, 7 and 12 wpi in the hepatic tissue by real-time quantitative polymerase chain reaction (qPCR).

**FINDINGS:**

Swiss mice showed 100% of survival, had lower parasite burden and intestinal eggs, while infected BALB/c and C57BL/6 presented 80% and 90% of survival, respectively, higher parasite burden and intestinal eggs. The three mouse lineages displayed distinct patterns in the expression of innate immune receptors, their adaptor molecules and cytokines, at 2 and 7 wpi.

**MAIN CONCLUSIONS:**

Our results suggest that the pathogenesis of *S. mansoni* infection is related to a dynamic early activation of innate immunity receptors and cytokines important for the control of developing worms.

Human schistosomiasis is a parasitic disease caused by trematode flukes of the family Schistosomatidae and genus *Schistosoma*. It is estimated that more than 250 million people worldwide are affected by Schistosomiasis^1^ causing intestinal or urogenital forms of the disease. *Schistosoma mansoni* is one of the parasites that cause intestinal Schistosomiasis. The acute phase is usually asymptomatic in individuals from endemic areas, however, during the chronic phase, approximately 50-60% of individuals have clinical manifestations and 10% develop the severe form of the disease, such as intestinal, hepatointestinal and hepatosplenic forms.^2^ ^,^ ^3^ ^,^ ^4^ ^,^ ^5^ The parasitic burden, immunity and other host intrinsic factors are major players in the severity of the clinical forms.^6^ ^,^ ^7^ ^,^ ^8^ People who eliminate large numbers of eggs in their faeces, have high worm numbers and more frequently develop the hepatosplenic form of Schistosomiasis.^9^ The parasite eggs play a key role in the Schistosomiasis pathology with the establishment of the granulomatous reaction in the liver, and breaking the intestinal epithelial barrier. However, other factors also contribute to the developing of severe clinical forms, such as nutritional status, immune response, host genetic background and comorbidities.^9^

Innate immunity represents the first line of defence against schistosomula after cercariae penetration into the skin.^10^ Innate immunity receptors have fundamental importance in initiating the immune response against the parasite and directing specific adaptive response, cytokines and antibodies production, and cell migration.^11^ ^,^ ^12^ Pattern recognition receptors (PRRs) play a key role in the orchestration of immune response by recognising pathogens-derived antigens during infection.^13^ Among the PRRs, toll-like receptors (TLRs) are described to be involved in the recognition of *S. mansoni* antigens, especially by TLR2, TLR3, TLR4 and TLR9.^14^ ^,^ ^15^ ^,^ ^16^ In general, the activation of TLRs leads to IL-12, TNF-α, pro-IL-1β and pro-IL-18 production.^17^ ^,^ ^18^ During the course of murine schistosomiasis, the activation of the NLRP3 and AIM2 inflammasomes is also described, resulting in the cleavage and release of the active IL-1β and IL-18.^19^ ^,^ ^20^

Although TLRs and NLRs have been described in the recognition of *S. mansoni* antigens,^21^ ^,^ ^22^ ^,^ ^23^ the differential expression of these PRRs during *S. mansoni* infection and its products in vertebrate hosts with distinct genetic background have not been completely determined yet. Here, we analysed the expression of innate molecules at transcript level correlating with *S. mansoni* infection course in three distinct mouse lineages: BALB/c, C57BL/6 and Swiss, at different time points after infection.

## MATERIALS AND METHODS

*Animals and ethics statement* - Specific-pathogen-free (SPF) BALB/c, C57BL/6 and Swiss Webster male mice, aged between six to eight weeks old, weighting about 25 g were used according to institutional ethical guidelines and the Ethics Committee on Animal Use (CEUA) of the Universidade Federal do Rio Grande do Norte (UFRN) under the protocol number 049/2014. All mice were housed in temperature-controlled rooms, with a 12 h/12 h dark/light cycle and receiving water and dry food *ad libitum*.

*Parasite and infection - S. mansoni* cercariae of the LE strain were maintained by successive passages in hamsters (*Mesocricetus auratus*) and laboratory reared and infected snails (*Biomphalaria glabrata*) at the Department of Parasitology (ICB/UFMG), as described by Pellegrino and Katz.^24^ A total of 90 mice were used in this study; the mice were distributed as follows: forty-five mice were randomly divided into three groups of 15 animals each (Balb/c, C57BL/6 and Swiss) and infected subcutaneously with approximately 30 cercariae of *S. mansoni* LE strain. Each mice group was appropriately euthanised at two, seven- and 12-weeks post infection (comprising five mice per group per time). Thirty mice were divided into the same three groups, with 10 animals each, to determine the survival rate after infection. Finally, 15 mice were used as uninfected control groups, being composed of 5 mice for BALB/c, C57BL/6 and Swiss each.

Parasitological parameters evaluation

*Survival rate* - BALB/c, C57BL/6 and Swiss mice were infected with the LE strain of *S. mansoni*, as previously mentioned, the clinical signs and death rate were monitored for up to 14 weeks after infection to determine the survival rate.

*Adult worms recovery in the liver portal system* - The recovery of parasites from portal hepatic system was performed at 7 and 12 wpi, as described by Pellegrino and Siqueira.^25^ Briefly, after euthanasia, mice had their peritoneal cavity opened to expose the intestine and to allow the identification of the portal vein. The worms were recovered by perfusion with 0.85% saline solution and transferred to a Petri dish to manual quantification and sex differentiation.

*Oogram* - Infected mice were submitted to euthanasia at 7 and 12 wpi and the intestines were collected to isolation of ileum, to allow the quantification of *S. mansoni* eggs per gram of intestinal tissue. This analysis was performed as described by Pellegrino and Faria.^26^ In summary, the intestinal fragment of the distal ileum (1 cm length) of each mouse was longitudinally opened and washed with 0.85% saline solution and weighed, then placed in a slip-covered slide. The number of eggs was manually determined using light microscope (using 10x objective), and classified as immature, mature, dead eggs, or eggshells, as described by Pellegrino et al.^27^ The relative number of eggs/gram of distal ileum was achieved using the formula, described by Mati and Melo^28^: eggs/gram of tissue = (total number of eggs in the tissue /tissue fragment weight in mg) x1000.

*Liver histology* - Fragments of hepatic tissue were fixed in 10% buffered formalin solution, dehydrated, cleared, and embedded in paraffin and cut into 4 μm-thick sections and stained by Hematoxylin-Eosin (HE) and slides mounted using Canada Balsam. Six 4μm-thick liver sections were examined for each animal, and 10 randomly chosen granulomas, with a miracidium-containing egg, were analysed under light microscopy (10x objective). The average sizes of the granulomatous lesions were obtained with the aid of an ocular millimetre grid and the longitudinal and transverse diameters of the granulomas were measured; the values were corrected based on the micrometric coefficient. The granuloma size (in mm) was expressed as the mean ± standard deviation.

Immunological parameters evaluation

*Real-time polymerase chain reaction (PCR)* - Liver fragments from *S. mansoni*-infected and non-infected animals were collected for total RNA extraction by using the SV Total RNA Isolation System (Promega, WI USA) according to the manufacturer’s instructions. Total RNA was quantified and used to produce cDNA with the High-Capacity cDNA Reverse Transcription Kit (Applied Biosystems, Warrington, UK), following the manufacturer’s specifications. cDNA was stored at -70ºC until real-time PCR reactions. Quantitative gene expression was determined from 2.5 ηg of cDNA per reaction by using the SYBR Green system and ABI Prism 7500 Fast Sequence Detection System machine (Applied Biosystems, Warrington, UK). The primers were synthesised using the Primer Express software (Applied Biosystems, USA) and are described at Supplementary data (Table). The mRNA expression levels of innate immune receptors (TLR1, TLR2, TLR3, TLR4, TLR5, TLR6, TLR7, TLR8, TLR9, NLRP1 and NLRP3), signalling molecules (MyD88, RIP-2, ASC and Caspase-1) and cytokines (IL-1β, IL-4, IL-6, IL-10, IL-12p35, IL-18 and TNF-α) was determined after normalisation with uninfected control groups (UC) and the expression of the constitutive β-actin gene using the 2^-ΔΔCt^ formula.

The analyses were carried out every five weeks, starting as soon as 2 wpi and finishing at 12 wpi.

*Statistical analysis* - Data are presented as the mean ± standard error of the mean (SEM). Kolmogorov-Smirnov and Shapiro-Wilk tests were used to verify parametric or non-parametric data distribution. Comparisons of parasite burden, parasite eggs number and mRNA expression levels between all the groups were performed by analysis of variance (ANOVA) followed by Tukey’s post-test for parametric data, or Kruskal-Wallis test followed by Dunn’s post-test for non-parametric data when necessary. Correlations were performed by the Pearson correlation coefficient. Differences between the groups were considered significant when p < 0.05. The analyses were performed using PRISM 8.0 software (GraphPad, CA, USA). Principal component analysis (PCA) was performed using ClustVis software.^29^

## RESULTS

**Fig. 1:**
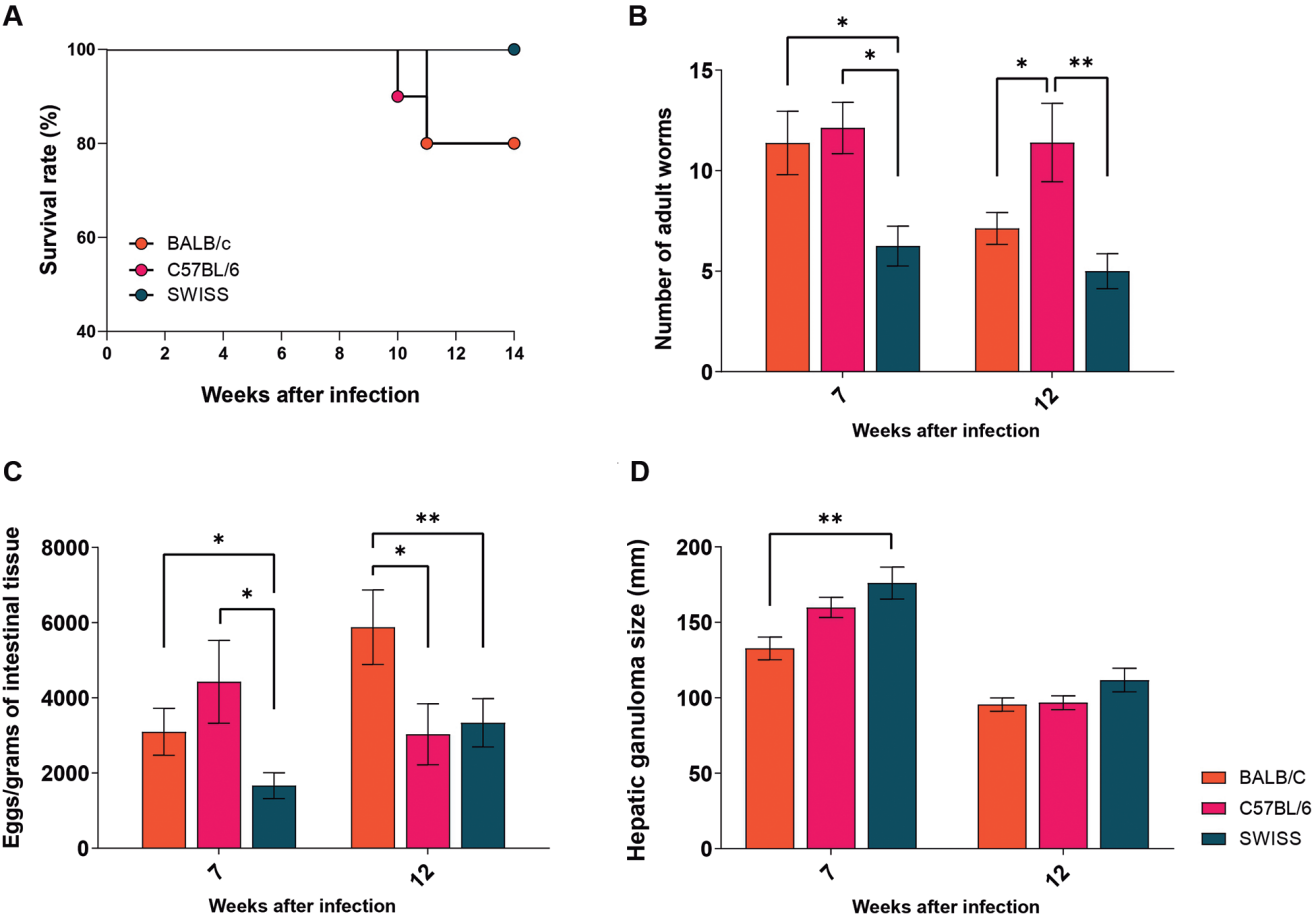
*Schistosoma mansoni-*infected Swiss mice harbour lower parasite burden than BALB/c and C57BL/6 infected mice at 7- and 12-weeks post-infection. (A) the survival rate of Balb/c (n = 10), C57BL/6 (n = 10) and Swiss (n = 10) mice, subcutaneously infected with 30 cercariae of *S. mansoni* LE strain, accompanied during 14 weeks after infection. (B) Number of adult parasites recovered from the hepatic portal system of BALB/c (n = 5), C57BL/6 (n = 5) and Swiss (n = 5). (C) eggs/gram of intestinal tissue, based on fragments of distal ileum of BALB/c (n = 5), C57BL/6 (n = 5) and Swiss (n = 5). (D) hepatic granuloma size of BALB/c (n = 5), C57BL/6 (n = 5) and Swiss (n = 5). The data represent two independent experiments, and the results are expressed as mean **±** standard error of the mean (SEM). *p < 0.05; **p < 0.01.

*Establishment of S. mansoni infection* - The course of *S. mansoni* infection in BALB/c, C57BL/6 and Swiss mice, was followed for the mortality, adult worm recovery, eggs/gram of intestinal tissue and hepatic granuloma size evaluation. After 14 wpi, infected Swiss mice displayed 100% survival; while 10% of C57BL/6 and 20% of BALB/c mice died between 10th and 11th wpi, respectively (Fig. 1A). Furthermore, Swiss mice displayed reduced numbers of adult worms in the portal system, when compared to BALB/c and C57BL/6 mice at 7 wpi (Fig. 1B). At 12 wpi, C57BL/6 exhibited higher numbers of recovered adult worms, when compared to BALB/c and Swiss mice (Fig. 1B). Reduced numbers of eggs/grams of distal ileum were found in Swiss mice at 7 wpi when compared to BALB/c and C57BL/6 mice; on the other hand, at 12 wpi BALB/c mice displayed increased number of eggs/grams of distal ileum when compared to Swiss and C57BL/6 mice (Fig. 1C).

Finally, regarding the granuloma size, Swiss mice presented larger hepatic granulomas when compared to BALB/c at 7 wpi, and no significant differences on the granuloma size were found between the analysed groups at 12 wpi (Fig. 1D).

**Fig. 2:**
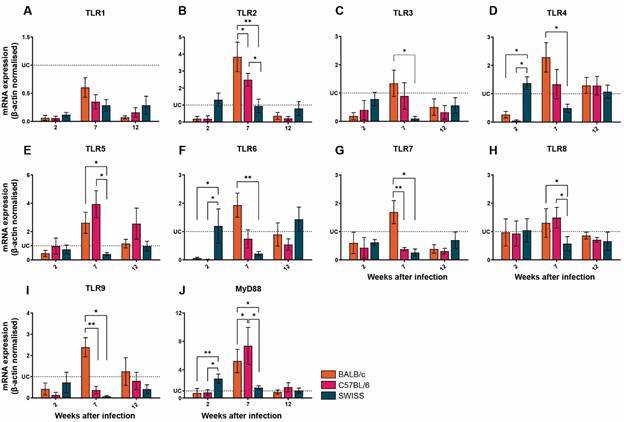
expression of Toll-like receptors in the liver of *Schistosoma mansoni-*infected mice dramatically differs considering mice lineage and time of infection. The mRNA expression levels of TLR1 (A), TLR2 (B), TLR3 (C), TLR4 (D), TLR5 (E), TLR6 (F), TLR7 (G), TLR8 (H), TLR9 (I), MyD88 (J) were determined by real-time polymerase chain reaction (PCR) in the liver from Balb/c (n = 5), C57BL/6 (n = 5) and Swiss (n = 5) mice subcutaneously infected with 30 cercariae of *S. mansoni* LE strain. The transcripts expression levels were normalised to uninfected controls (dotted lines) and by the expression level of the β-actin housekeeping gene. The data represent two independent experiments, and the results are expressed as the means ± standard error of the mean (SEM). *p < 0.05; **p < 0,01. UC: uninfected controls (n = 5).

**Fig. 3:**
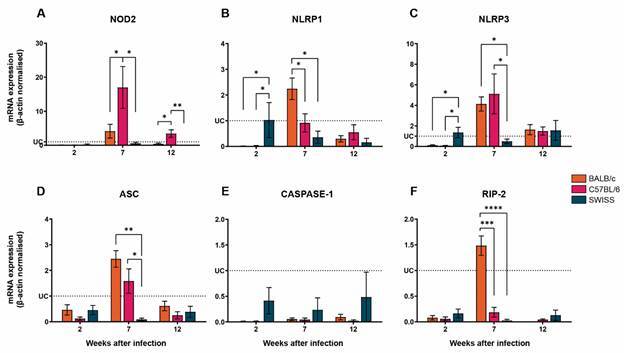
NLRP3 and ASC mRNA are upregulated in the liver of *Schistosoma mansoni-*infected BALB/c and C57BL/6 mice at 7-weeks post-infection. The mRNA expression levels of NOD2 (A), NLRP1 (B), NLRP3 (C), ASC (D), Caspase-1 (E) and RIP-2 (F) were determined by real-time polymerase chain reaction (PCR) in the liver from *S. mansoni*-infected BALB/c (n = 5), C57BL/6 (n = 5) and Swiss (n = 5) mice. The expression levels were normalised to the expression level of the β-actin housekeeping gene. The transcripts expression levels were normalised to uninfected controls (dotted lines) and by the expression level of the β-actin housekeeping gene. The data represent two independent experiments, and the results are expressed as the means ± standard error of the mean (SEM). *p < 0.05; **p < 0.01; ***p < 0.001; ****p < 0.0001. UC: uninfected controls (n = 5).

*Early activation of innate immune receptors differs according to the mice background* - In order to investigate the profile of innate immune response activation during *S. mansoni* infection, we performed a kinetics of the expression of genes related to innate immune receptors, known to play a very crucial role in initiating and modulating immunological responses. By analysing the expression level of TLR1 - 9 and the adaptor molecule MyD88 in each mouse lineage, it was found that *S. mansoni-*infected BALB/c mice displayed higher mRNA expression of TLR1-7, -9 and MyD88 at 7 wpi [Supplementary data (Fig. 1A)]. C57BL/6 mice displayed higher mRNA expression of TLR2, -5 and MyD88 at 7 wpi, and TLR4 and six were highly expressed at 7 and 12 wpi [Supplementary data (Fig. 2A)]. Contrasting results were found for infected Swiss mice, since higher mRNA expression for TLR3, -4 and MyD88 was found at 2 wpi; in addition, TLR6 were found to be upregulated at 12 wpi, although no significant differences were found when comparing with 2 wpi [Supplementary data (Fig. 3A)].

After analysing the expression profile within each mouse lineage at different time points, we further analysed the differences on gene expression between those lineages. We observed that the *S. mansoni* infection downregulates TLR1 mRNA expression at all time points evaluated independently of the mice lineages, compared to uninfected animals (Fig. 2A). Swiss mice showed higher mRNA expression for TLR4, TLR6 and Myd88 at 2nd wpi, when compared to the BALB/c and C57BL/6, which expressed similar level of these transcripts (Fig. 2D, F, J). Interestingly, seven weeks post infection, this pattern shifted, as BALB/c and C57BL/6 upregulated the expression of TLR2, TLR5, TLR8, TLR9, and MyD88 mRNA (Fig. 2B, E, H, J). Upregulation of TLR3, TLR4, TLR6, TLR7, and TLR9 mRNA was observed only among infected BALB/c when compared to Swiss mice (Fig. 2C, D, F, G, I) at 7 wpi. Although no significant differences in the transcript expressions analysed were observed when comparing the groups at 12 wpi (Fig. 2), TLR1, TLR2, TLR3, TLR7 and TLR8 were found to be downregulated in all three mouse lineages at 12 wpi, when compared to the uninfected control.

In an attempt to verify if the differential expression of another PRR family could also be involved in the activation of innate immune response during *S. mansoni* infection, we analysed the expression of inflammasome-involved molecules. Firstly, we evaluated the gene expression within each mouse lineage. It was found that mRNA transcripts for NOD2, NLRP1, NLRP3, and ASC were highly expressed, followed by high RIP-2 expression at 7 wpi in *S. mansoni-*infected BALB/c mice [Supplementary data (Fig. 1B)]. C57BL/6 infected mice displayed upregulated expression of NOD2, NLRP3 and ASC at 7 wpi; NLRP1 although downregulated during the infection course, it was found with higher mRNA expression at 7 wpi than at 2 wpi [Supplementary data (Fig. 2B)]. Interestingly, although no significant differences on the level of mRNA expression were found within the infected Swiss mice in the three time points analysed, the mRNA transcripts were downregulated [Supplementary data (Fig. 3B)].

Furthermore, we analysed the differences in the mRNA transcripts expression between the three mouse lineages. At 2 wpi, no significant alterations were observed in the levels of NOD2, ASC, Caspase-1 and RIP-2 mRNAs, considering the three mouse lineages (Fig. 3A, D-F). On the other hand, at the same time point, infected Swiss mice, showed higher levels of transcripts to NLRP1, NLRP3 (Fig. 3B, C). After 7 wpi, BALB/c mice displayed higher expression of NLRP1 and RIP-2 mRNA, when compared to C57BL/6 and Swiss mice (Fig. 3C, D, F); at this time point, NLRP3 and ASC were found to be highly expressed by both BALB/c and C57BL/6 mice (Fig. 3C, D). At 12 wpi, the levels of NOD2 were upregulated only in C57BL/6-infected mice (Fig. 3C). No significant changes between the three groups were found for NLRP1, NLRP3, ACS, Caspase-1 or RIP-2 at 12 wpi (Fig. 3B-F).

Together, these data indicate that the expression of specific innate immune receptors and their adaptor molecules can be differentially activated according to the host background and to the time of *S. mansoni* infection.

*Inflammatory and anti-inflammatory cytokines are differentially expressed according to the time of S. mansoni infection* - The activation of PRRs can be indirectly assessed by the measurement of downstream products as the transcripts of innate cytokines. In this sense, the expression of pro-IL-1β, pro-IL-18, IL-6, IL-10, IL-12p35, IL-4 and TNF-α was assessed in the same kinetics as described for the PRRs mRNA expression analysis. Firstly, we analysed the gene expression within each mouse lineage. In *S. mansoni-*infected BALB/c mice, the levels of TNF-α mRNA expression was higher at 7 wpi when compared to 2 wpi, although no differences were found when comparing with 12 wpi. In addition, pro-IL-1β was found to be upregulated only at 12 wpi, and IL-4 was found to be highly expressed at both 7 and 12 wpi [Supplementary data (Fig. 1C)]. In C57BL/6 infected mice, TNF-α mRNA was found to be upregulated only at 7 wpi, while pro-IL-1β mRNA was found to be upregulated at all time points analysed. The transcripts for IL-18 and IL-12p35 were found to be negatively regulated at the three time points, when compared to the uninfected group, however at 7 wpi infected C57BL/6 mice displayed higher levels of mRNA for IL-18 and IL-12p35 when compared to the 2nd and 12th wpi [Supplementary data (Fig. 2C)]. In C57BL/6 mice, the expression level of IL-4 mRNA was found to be significantly higher at 12 wpi when compared to the uninfected group [Supplementary data (Fig. 2C)]. Finally, when analysing the mRNA expression levels in *S. mansoni-*infected Swiss mice we found that most of the cytokines transcripts were downregulated and with no differences between the three time points. However, pro-IL-1β was found to be upregulated in all time points, and IL-4 mRNA displayed higher expression at 7 and 12 wpi compared to uninfected control group [Supplementary data (Fig. 3C)].

**Fig. 4:**
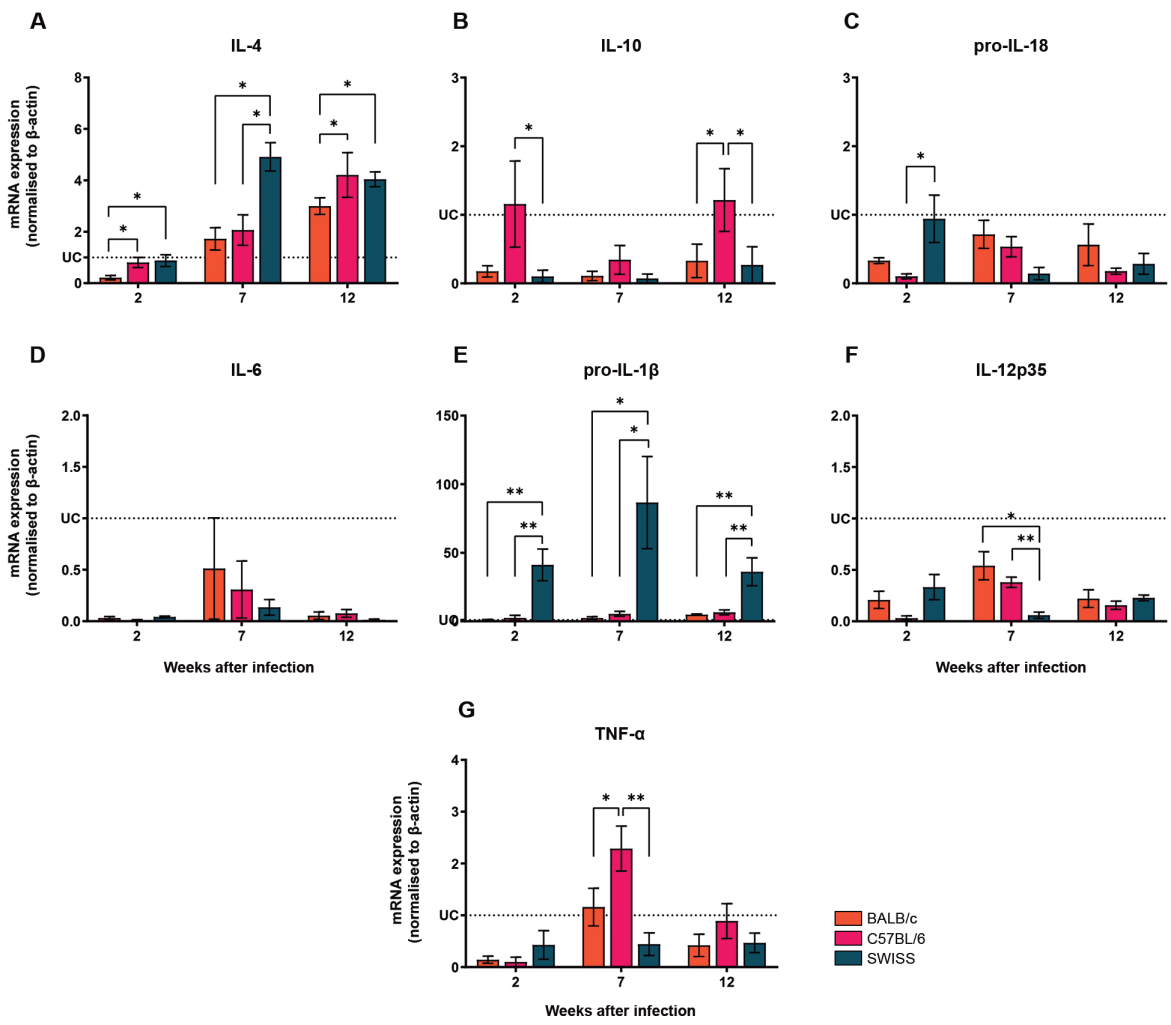
Swiss mice displayed higher levels of IL-4 and pro-IL-1β in the liver after *Schistosoma mansoni* infection. The mRNA expression levels of IL-4 (A), IL-10 (B), pro-IL-18 (C), IL-6 (D), pro-IL-1β (E), IL-12p35 (F) and TNF-α (G) were determined by real-time polymerase chain reaction (PCR) in the liver of *S. mansoni-*infected BALB/c (n = 5), C57BL/6 (n = 5) and Swiss (n = 5) mice. The transcripts expression levels were normalised to uninfected controls (dotted lines) and by the expression level of the β-actin housekeeping gene. The data represent two independent experiments, and the results are expressed as the means ± standard error of the mean (SEM). *p < 0.05; **p<0.01. UC: uninfected controls (n = 5).

Now, considering the differences between the linages, when assessed at the different time points, the levels of IL-4 mRNA were found to be differentially expressed among the three mouse lineages. At 2 wpi, C57BL/6 and Swiss mice displayed higher levels of IL-4 transcripts, when compared to BALB/c mice. Infected Swiss mice displayed significantly higher levels of IL-4 mRNA at 7 wpi, when compared to BALB/c and C57BL/6 mice, and remained elevated at 12 wpi with transcript levels similar to C57BL/6 group (Fig. 4A).

The levels of IL-10 mRNA were distinctly regulated at the different time points; it was found an upregulation of this transcript at 2 and 12 wpi in infected C57BL/6 mice, when compared to either Swiss, BALB/c or uninfected groups (Fig. 4B). In addition, Swiss mice showed higher levels of pro-IL-18 at week 2, when compared to BALB/c and C57BL/6 mice (Fig. 4C); the levels of IL-6 mRNA were found to be downregulated at 2 and 12 wpi for all infected mouse groups, when compared to the uninfected control (Fig. 4D). Interestingly, *S. mansoni*-infected Swiss mice showed higher expression of pro-IL-1β mRNA in the liver at 2, 7 and 12 wpi, when compared to BALB/c and C57BL/6 mice (Fig. 4E); although IL-12p35 was shown to be downregulated, when compared to uninfected controls, BALB/c and C57BL/6 mice displayed higher levels of this transcript only at 7 wpi (Fig. 4F). Finally, infection by *S. mansoni* led to an increase of TNF-α mRNA in the livers of C57BL/6 mice at 7 wpi, compared to Swiss and BALB/c mice (Fig. 4G).

**Fig. 5:**
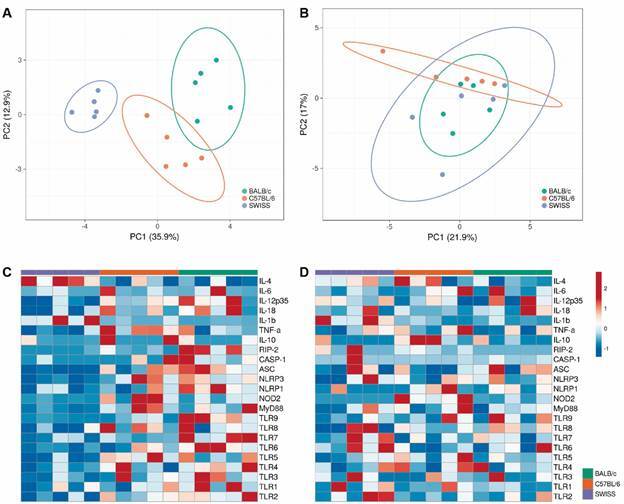
principal component analysis revealed that *Schistosoma mansoni*-infected Swiss mice represent a completely distinct cluster from infected BALB/c and C57BL/6. Principal component analysis (PCA) were performed considering the expression levels of all immunological markers evaluated in this study. (A) PCA using combined data of the expression levels of toll-like receptors (TLRs), inflammasome-related molecules and cytokines, evidencing the cluster organisation between *S. mansoni*-infected BALB/c (n-5), C57BL/6 (n = 5) and Swiss (n = 5) at 7-weeks post-infection. (B) PCA using combined data of the expression levels of TLRs, inflammasome-related molecules and cytokines, evidencing the cluster organisation between *S. mansoni*-infected BALB/c (n-5), C57BL/6 (n = 5) and Swiss (n = 5) at 12-weeks post-infection. (C) Heatmap of the expression levels of TLRs, inflammasome-related molecules and cytokines mRNAs at 7 wpi. (D) Heatmap of the expression levels of TLRs, inflammasome-related molecules and cytokines mRNAs at 12 wpi.

These results indicate that the *S. mansoni* infection modulates the expression of specific cytokines, especially in the initial stage of infection, which overlap with the kinetics observed in several PRRs. This event might be necessary to control of larval forms of the parasite, leading, in the end, to a lower parasite load and animal survival as observed among infected Swiss mice, with high IL-4 expression the during late acute phase of infection (7th week), which is important to control adult forms of the parasite and by down modulating Th1 response. The dynamic interplay of PRRs, their adapter molecules and cytokines among the different mouse strains used in this study highlighted the differences on the immune response activation as shown by the PCA analysis, in which during the 7th wpi infected Swiss mice represent a completely distinct cluster from BALB/c and C57BL/6 infected mice (Fig. 5A). The cluster differentiation observed at this time point dramatically changes when the infection reach week 12, where no clustering differences are found (Fig. 5B). This analysis can be explained by the changes in the expression of the specific markers analysed, at 7 wpi (Fig. 5C) and 12 wpi (Fig. 5D).

Aiming to correlate the expression of innate immunity cytokines transcripts with the course of infection by *S. mansoni* in different mouse strains, we crossed data of the number of eggs per gram of the distal ileum or the number of adult worms recovered from the hepatic perfusion with the pro-IL-1β and TNF-α mRNA expression at weeks 7 and 12 post infection. A positive correlation was observed, at 7th and 12th week, between TNF-α mRNA expression and the total number of adult *S. mansoni* parasites in the hepatic portal system and eggs/gram of intestine tissue [Supplementary data (Fig. 4A-D)]. These results indicate that the elevated hepatic expression of TNF-α in the chronic phase of infection by *S. mansoni* can be a factor associated with susceptibility to the experimental infection in mice. A negative correlation was observed between the expression of pro-IL-1β mRNA with the number of parasites in the portal system at 7 and 12 weeks after infection [Supplementary data (Fig. 5A, B)]. Also, a negative correlation was observed between the pro-IL-1β expression with the *S. mansoni* eggs/grams at 12 weeks after infection [Supplementary data (Fig. 5C, D)], suggesting the protective role of IL-1β in experimental infection with *S. mansoni.* Regarding the expression of IL-4 mRNA, it was found a negative correlation between the expression levels of this transcript and adult worms recovered from the hepatic portal system at 7 wpi [Supplementary data (Fig. 6A)]. No significant correlations were found for the same parameter at 12 wpi [Supplementary data (Fig. 6B)], nor for the relationship between the levels of IL-4 mRNA and the number of eggs/grams of intestinal tissue at 7 and 12 wpi [Supplementary data (Fig. 6C, D).

## DISCUSSION

The PRRs expression and innate cytokine production contribute to the modulation of immune responses triggered by *S. mansoni* infection.^23^ ^,^ ^30^ Here, we evaluated the differential mRNA expression of several innate immunity molecules in the main *S. mansoni* target organ, the liver, of BALB/c, C57BL/6 and Swiss mouse lineages*.*

The course of *S. mansoni* infection can be assessed by adult helminths in the hepatic portal system, number and viability of released eggs, formation of liver granulomas and mortality rate.^31^ ^,^ ^32^ ^,^ ^33^ ^,^ ^34^ ^,^ ^35^ ^,^ ^36^ We showed that 100% of Swiss animals survived after infection with *S. mansoni* and they had lower worms than BALB/c and C57BL/6 mice, which presented 80% and 90% of survival, respectively. Literature data have been variable about mortality, adult worm recovery and the intestinal number of eggs described in BALB/c and C57BL/6 mice experimentally infected with different inoculum and several *S. mansoni* strains.^33^ ^,^ ^35^ ^,^ ^37^ ^-^ ^40^ In the present study, BALB/c and C57BL/6 mice infected by *S. mansoni* had more eggs/gram of intestine tissue and started to die between 10th and 11th week after infection. An important cause of pathology is associated with the host’s immune reaction against the egg antigens, mainly in the liver tissue, with maximum intensity in the 8th week after infection. After the eighth week of infection, there is a modulation of the immune reaction and the inflammatory foci around eggs become progressively smaller.^40^ ^,^ ^41^ ^,^ ^42^ ^,^ ^43^ ^,^ ^44^

Our study showed that in the early stage of *S. mansoni* infection (two weeks post infection) Swiss mice showed higher expression of TLR4, TLR6 and Myd88 mRNA in the liver than BALB/c and C57BL/6. Toll-like receptors have been described in the recognition of *S. mansoni* molecular patterns. TLR2 recognises the lysophosphatidylserine antigen that constitutes the soluble egg antigen (SEA),^18^ TLR3 is involved in the recognition of schistosome egg double-stranded RNA,^45^ TLR4 recognises the lacto-N-fucopentaose III (LNFPIII) component present in SEA, larvae and adult worms,^46^ and SEA also enhances TLR9 activation in macrophages.^47^ It is important to highlight the kinetics observed in the expression of TLR in the course of infection. In our study, we found that at 12 wpi, no significant differences were maintained in the mRNA expression levels, although most of TLR were found to be downregulated at this time point for the three mouse lineages analysed here. In a recent study, *S. mansoni*-infected Swiss mice displayed differential expression of TLR2, TLR3, TLR4, TLR7 and TLR9 mRNA in the liver, spleen and large intestine at approximately 12 wpi; the liver presented with the highest expression of these PRR, especially TLR4 and TLR3.^23^

Besides TLRs, *S. mansoni* infection also induced higher levels of inflammasome involved-molecules as NLRP1, NLRP3 and Caspase-1 in the second week of infection. The parasite is known to trigger apoptosis, mitochondrial dysfunction and ROS production in mouse livers, which activates NLRP3 inflammasome.^19^ ^,^ ^20^ NLRP1 and NLRP3 inflammasome contribute to the activation of inflammatory Caspase-1 that results in the proteolytic processing and secretion active forms of IL-1β and IL-18 cytokines.^48^ AIM2 inflammasome is also described to be activated during *S. mansoni* infection by soluble antigen eggs and potentially triggered by the parasite dsDNA in the cells.^19^ In our study, we showed that the molecules involved in the inflammasome pathway were differentially expressed between the mouse lineages; however, BALB/c mice displayed higher expression of NLRP1, NLRP3 and ASC at 7 wpi. In a previous study, it was reported that infection by *S. japonicum* in BALB/c mice led to increased formation of NLRP3 inflammasome in the liver at approximately 6 wpi, with this pathway being directly related to the fibrotic process in the liver triggered during Schistosomiaisis.^49^

As a consequence of several TLRs induction and activation during the period of larval migration in Swiss mice, pro-IL-1β levels were found to be higher in Swiss mice than BALB/c and C57BL/6. This event might be involved in the control of *S. mansoni* larval forms by classical macrophages-dependent mechanisms.^14^ ^,^ ^46^ ^,^ ^50^ ^,^ ^51^ ^,^ ^52^ IL-1β can increase the expression of adhesion molecules in endothelial cells, induce different chemokines (which promote cell infiltration and inflammation) in addition to activate the inducible nitric oxide synthase (iNOS) enzyme.^53^ The nitric oxide production can be induced by IL-1β/α, together with IFN-γ and/or TNF-α, resulting in the death of newly transformed larvae.^54^ Here, we observed a negative correlation between liver pro-IL-1β mRNA expression and the parasite burden, indicating the protective function of this cytokine. Thus, high activation of innate immunity receptors (TLR4, TLR6) and inflammatory cytokines (such as pro-IL-1β) in Swiss mice during the acute phase of infection may contribute to the low parasite burden.

The mortality of BALB/c and C57BL/6 *S. mansoni*-infected mice at the beginning of the chronic phase might be related to the activation of innate immunity receptors (TLRs and NLRs) at the week 7 post-infection. Here, we observed higher TLR2, TLR4, TLR5, TLR6, TLR8, TLR9, Myd88, NLRP3, ASC, and TNF-α mRNA expression at seven weeks after infection by *S. mansoni* in BALB/c and C57BL/6 mice compared to Swiss mice. Moreover, there is a positive correlation between TNF-α mRNA expression with the parasite burden at week 7 and 12 post-infection. It was previously shown that TNF-α plays a complex role during *S. mansoni* infection in mice. During experimental mice infection, this cytokine was able to play a protective role against hepatocellular damage and cachexia in C57BL/6 mice,^55^ in addition to be related to the eggs deposition during infection in SCID mice.^56^ Considering the role of this cytokine in the macrophage infiltration and activity, in addition to the granulomatous reaction in distinct granuloma-forming infections,^57^ we can argue that the kinetics observed in the expression of TNF-α and eggs deposition in C57BL/6 reflects the dynamic immunological balance triggered during *S. mansoni* infection, however more studies are still necessary to evaluate specific pathways and cellular roles during the establishment of *S. mansoni*-derived immunopathogenesis.

On the other hand, Swiss mice presented higher IL-4 mRNA expression in the hepatic tissue, in addition to higher hepatic granuloma size, at seven weeks after infection. IL-4 production induces CD4^+^ T cells differentiation in Th2 profile, leading to IL-4, IL-5, and IL-13 production. The chronic granulomatous response against the egg is mainly orchestrated by Th2-differentiated CD4^+^ T cells. A Th2-derived multicellular granulomas formation surrounding the *S. mansoni* eggs is important for the containment of egg antigens, avoid excessive inflammation, fibrosis and tissue damage^58^ and also contributes to killing adult *S. mansoni* parasites.^59^ By contrast, the Th1 response is ineffective in wound healing and collagen deposition, enabling the transit of eggs through the intestinal wall, which leads to the loss of the intestinal epithelial layer integrity and an influx of bacteria from the lumen, that can cause septicemia and death.^60^ The excessive Th1 response generates granulomas with a microenvironment rich of M1 macrophages and their respective inflammatory mediators, while few eosinophils are present, which may contribute to the liver and intestinal inflammation and mortality. In this scenario, it would be of great importance to analyse the expression profile of the remaining signature cytokines of the Th1 and Th2 profiles, to address a clearer vision of the inflammatory pathway during this helminth infection in further studies.

Altogether, our results indicate that *S. mansoni* infection in mice with distinct genetic backgrounds leads to differential activation of important innate immunity molecules, including TLRs, inflammasome-related molecules and cytokines transcripts. We showed that *S. mansoni-*infected Swiss mice represents a completely distinct group from BALB/c and C57BL/6 infected mice, regarding the levels of expression of the analysed markers. The kinetics of immune activation during *S. mansoni* infection dramatically changes from 7 wpi to 12 wpi, with the inflammatory parameters being associated with the parasite burden intensity among the infected animals.
