# Supplementary material for: Innate immune receptors are differentially expressed in mice during experimental Schistosoma mansoni early infection
Source: Mem Inst Oswaldo Cruz. 2024 Jun 17;119:e240013. doi: 10.1590/0074-02760240013 (PMC11182339; doi:10.1590/0074-02760240013)
Supplement: Supplementary file 1 [file 1678-8060-mioc-119-e240013-s.pdf]

TABLE

Sequence of the used specific starters in the reactions of polymerase chain reaction (PCR) in real time

Primers	Sense	Antisense
β-actin	AGGCAAACCGTGAAAAGATG	CTGTGGTACGACCAGAGGAATA
TLR1	TCTCTTCGGCACGTTAGC	CGTAAGAAATAAGAGCAGCCC
TLR2	CGAGTGGTGCAAGTACG	GGTAGGTCTTGGTGTTCATTATC
TLR3	GGTGGTCCCGTTAATTTCCT	CCCGAAAACATCCTTCTCAA
TLR4	CCTCTGCCTTCACTACAGAGACTTT	TGTGGAAGCCTTCCTGGATG
TLR5	CGCACGGCTTTATCTTCTCC	GGCAAGGTTCAGCATCTTCAA
TLR6	CCGGTGGAGTACCTCAAT	TCAGCAAACACCGAGTATAGC
TLR7	TGGAAATTTTGGACCTCAGC	TTGCAAAGAAAGCGATTGTG
TLR8	CACGTGTGACATAAGTGATTTTCG	TTTGATCCCCAGGATTGGAA
TLR9	CTGCCGCTGACTAATCTG	CTGAAATTGTGGCCTATACCC
NLRP1	TGGCACATCCTAGGGAAATC	TCCTCACGTGACAGCAGAAC
NLRP3	AGCCTTCCAGGATCCTCTTC	GGGCAGCAGTTTCTTTC
NOD2	CTTCATTTGGCTCATCCGTAG	CTGGAGATGTTGCAGTACAAAG
MyD88	TGATGCGGAGCCAGATT	GAGGAGGCATGTGTGTACT
ASC	AAGCTGCTGACAGTGCAAC	GCCACAGCTCCAGACTCTTC
Caspase-1	AGATGGCACATTTCCAGGAC	CCTCCAGCAGCAACTTC
pro-IL-1β	GCAACTGTTCCTGAACTCAACT	ATCTTTTGGGGTCCGTCAACT
RIP-2	GGAGGAACAATCATCTATATGCC	ATGATCTGCAAAGGATTGGT
IL-6	CCATCCAGTTGCCTTCTTG	AAGTGCATCATCGTTGTTCATAC
IL-4	CTGACGGCACAGAGCTATTGA	TATGCGAAGCACCTTGGAAGC
IL-10	TGGACAACATACTGCTAACC	GGATCATTTCCGATAAGGCT
IL-12p35	TCTCTGGACCTGCCAGGTGT	CCTGTTGATGGTCACGACGCG
pro-IL-18	GTGAAGTAAGAGGACTGGCTGTG	TTTTGGCAAGCAAGAAAGTGT
TNF-α	TGTGCTCAGAGCTTTCAACAA	CTTGATGGTGGTGCATGAGA

cc BY

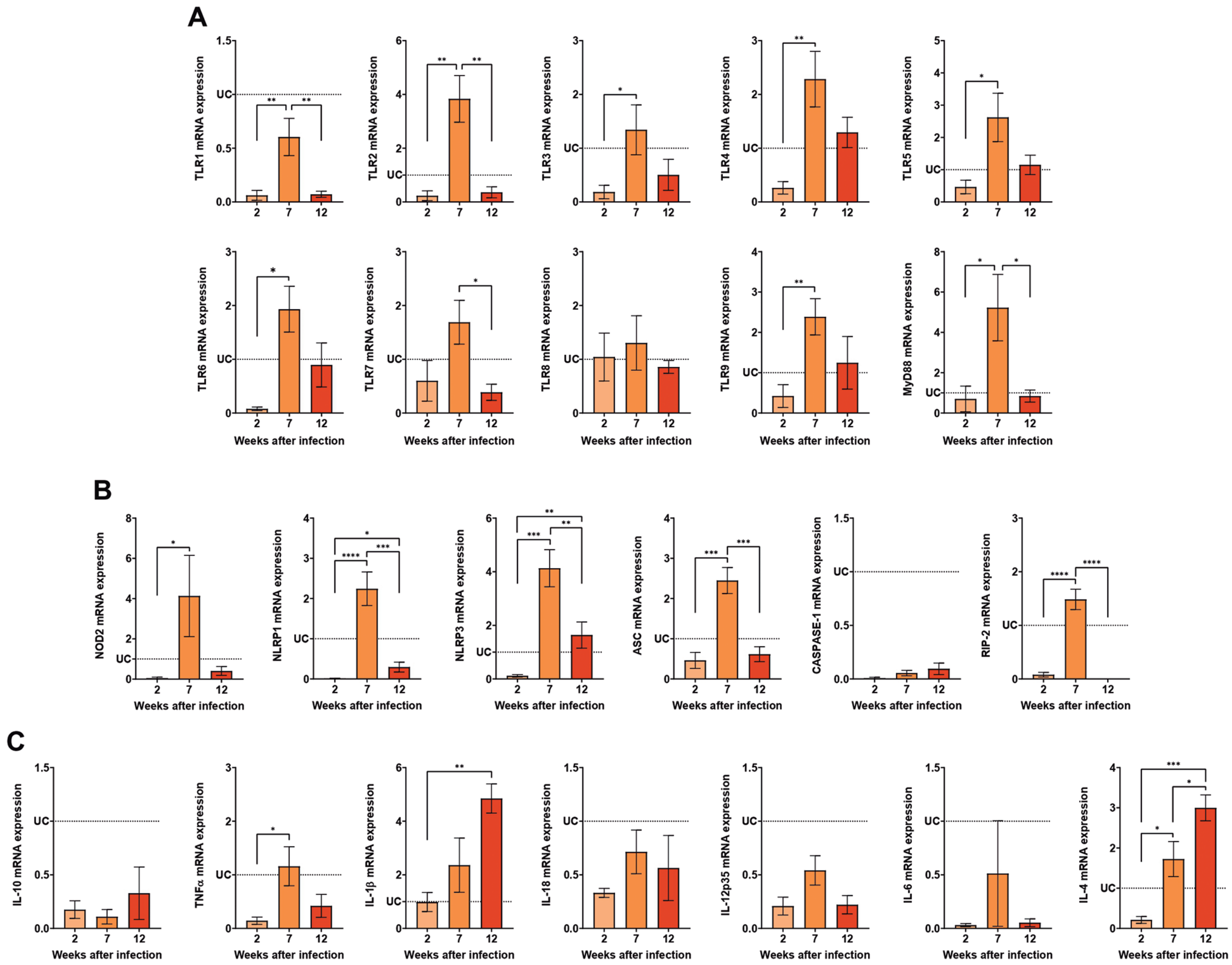

Fig. 1: expression levels of mRNA transcripts for Toll-like receptors, inflammasome-associated molecules and cytokines in the liver of *Schistosoma mansoni*-infected BALB/c mice. Expression levels of mRNA transcripts for TLR1, TLR2, TLR3, TLR4, TLR5, TLR6, TLR7, TLR8, TLR9, MyD88 (A). Expression levels of mRNA transcripts for NOD2, NLRP1, NLRP3, ASC, Caspase-1 and RIP-2 (B). Expression levels of mRNA transcripts for IL-4, IL-10, pro-IL-18, IL-6, pro-IL-1β, IL-12p35 and TNF-α (C). The expression level of all transcripts were determined by real-time polymerase chain reaction (PCR) in the liver from Balb/c (n = 5) mice subcutaneously infected with 30 cercariae of *S. mansoni* LE strain. The expression levels were normalised to uninfected controls (dotted lines) and by the expression level of the β-actin housekeeping gene. The data represent two independent experiments, and the results are expressed as the means ± standard error of the mean (SEM). *p < 0.05; **p < 0.01; ***p < 0.001; ****p < 0.0001. UC: uninfected controls (n = 5).

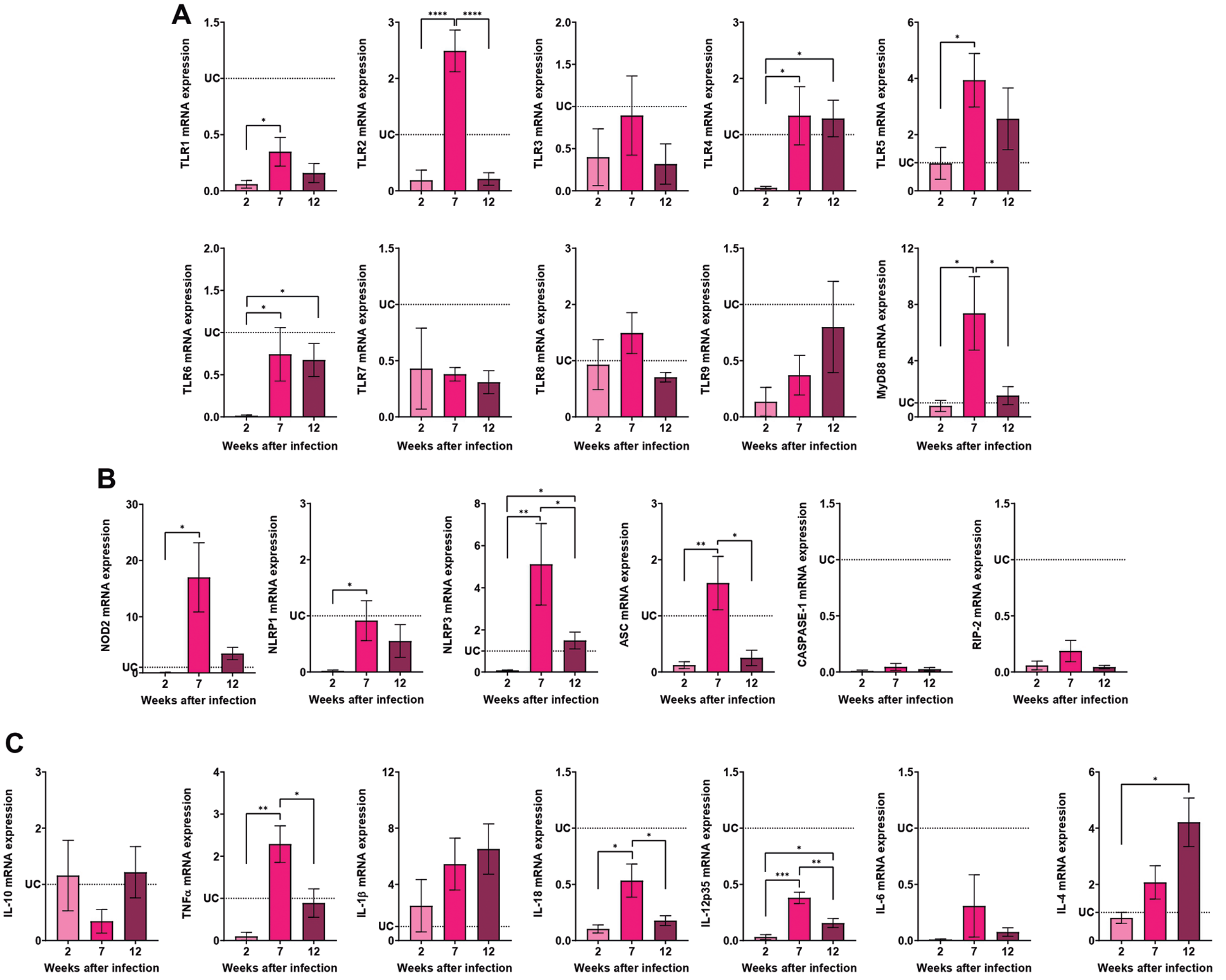

Fig. 2: expression levels of mRNA transcripts for Toll-like receptors, inflammasome-associated molecules and cytokines in the liver of *Schistosoma mansoni*-infected C57BL/6 mice. Expression levels of mRNA transcripts for TLR1, TLR2, TLR3, TLR4, TLR5, TLR6, TLR7, TLR8, TLR9, MyD88 (A). Expression levels of mRNA transcripts for NOD2, NLRP1, NLRP3, ASC, Caspase-1 and RIP-2 (B). Expression levels of mRNA transcripts for IL-4, IL-10, pro-IL-18, IL-6, pro-IL-1β, IL-12p35 and TNF-α (C). The expression level of all transcripts were determined by real-time polymerase chain reaction (PCR) in the liver from C57BL/6 (n = 5) mice subcutaneously infected with 30 cercariae of *S. mansoni* LE strain. The expression levels were normalised to uninfected controls (dotted lines) and by the expression level of the β-actin housekeeping gene. The data represent two independent experiments, and the results are expressed as the means ± standard error of the mean (SEM). *p < 0.05; **p < 0.01; ***p < 0.001; ****p < 0.0001. UC: uninfected controls (n = 5).

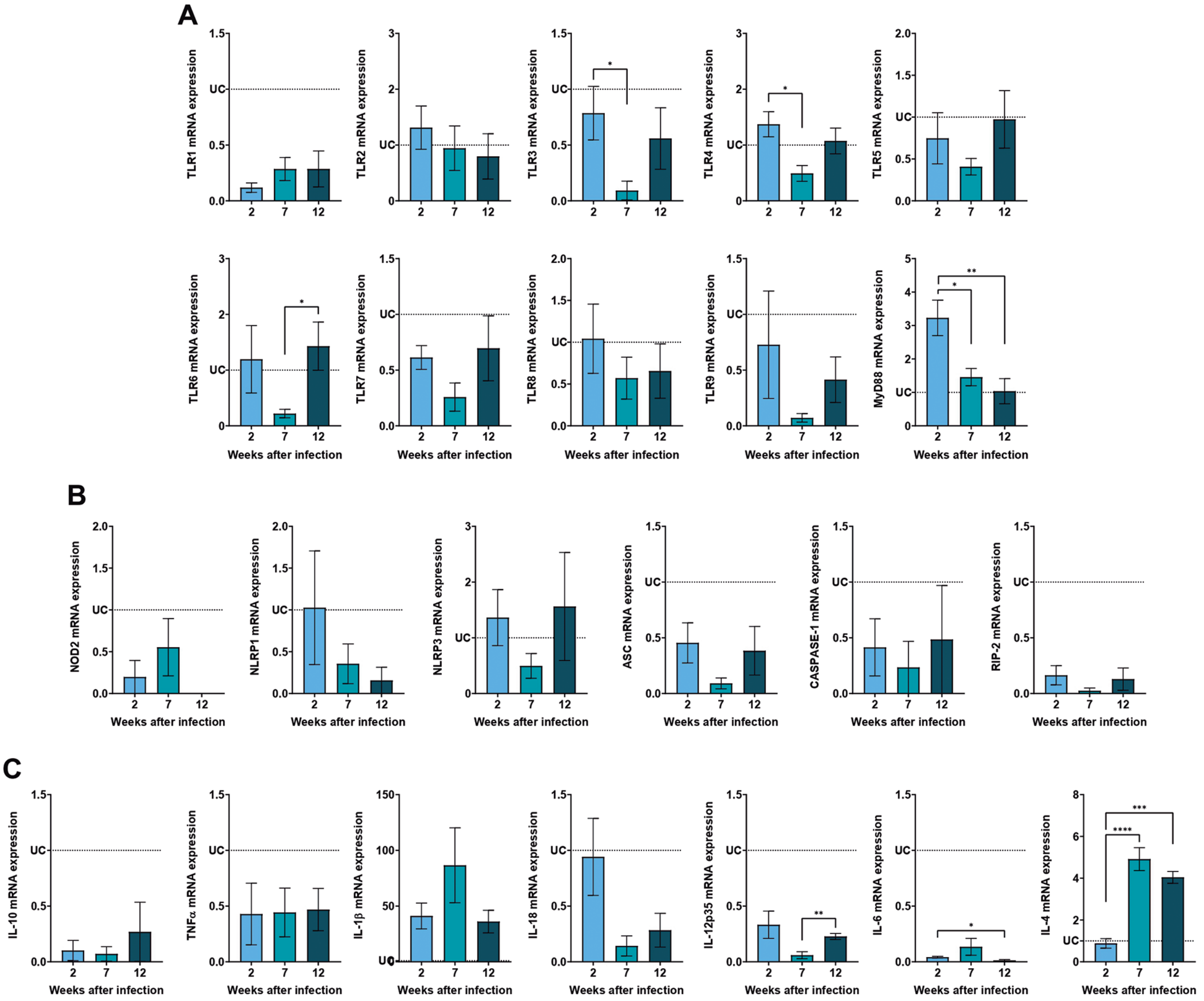

Fig. 3: expression levels of mRNA transcripts for Toll-like receptors, inflammasome-associated molecules and cytokines in the liver of *Schistosoma mansoni*-infected Swiss mice. Expression levels of mRNA transcripts for TLR1, TLR2, TLR3, TLR4, TLR5, TLR6, TLR7, TLR8, TLR9, MyD88 (A). Expression levels of mRNA transcripts for NOD2, NLRP1, NLRP3, ASC, Caspase-1 and RIP-2 (B). Expression levels of mRNA transcripts for IL-4, IL-10, pro-IL-18, IL-6, pro-IL-1β, IL-12p35 and TNF-α (C). The expression level of all transcripts were determined by real-time polymerase chain reaction (PCR) in the liver from Swiss (n = 5) mice subcutaneously infected with 30 cercariae of *S. mansoni* LE strain. The expression levels were normalised to uninfected controls (dotted lines) and by the expression level of the β-actin housekeeping gene. The data represent two independent experiments, and the results are expressed as the means ± standard error of the mean (SEM). *p < 0.05; **p < 0.01; ***p < 0.001; ****p < 0.0001. UC: uninfected controls (n = 5).

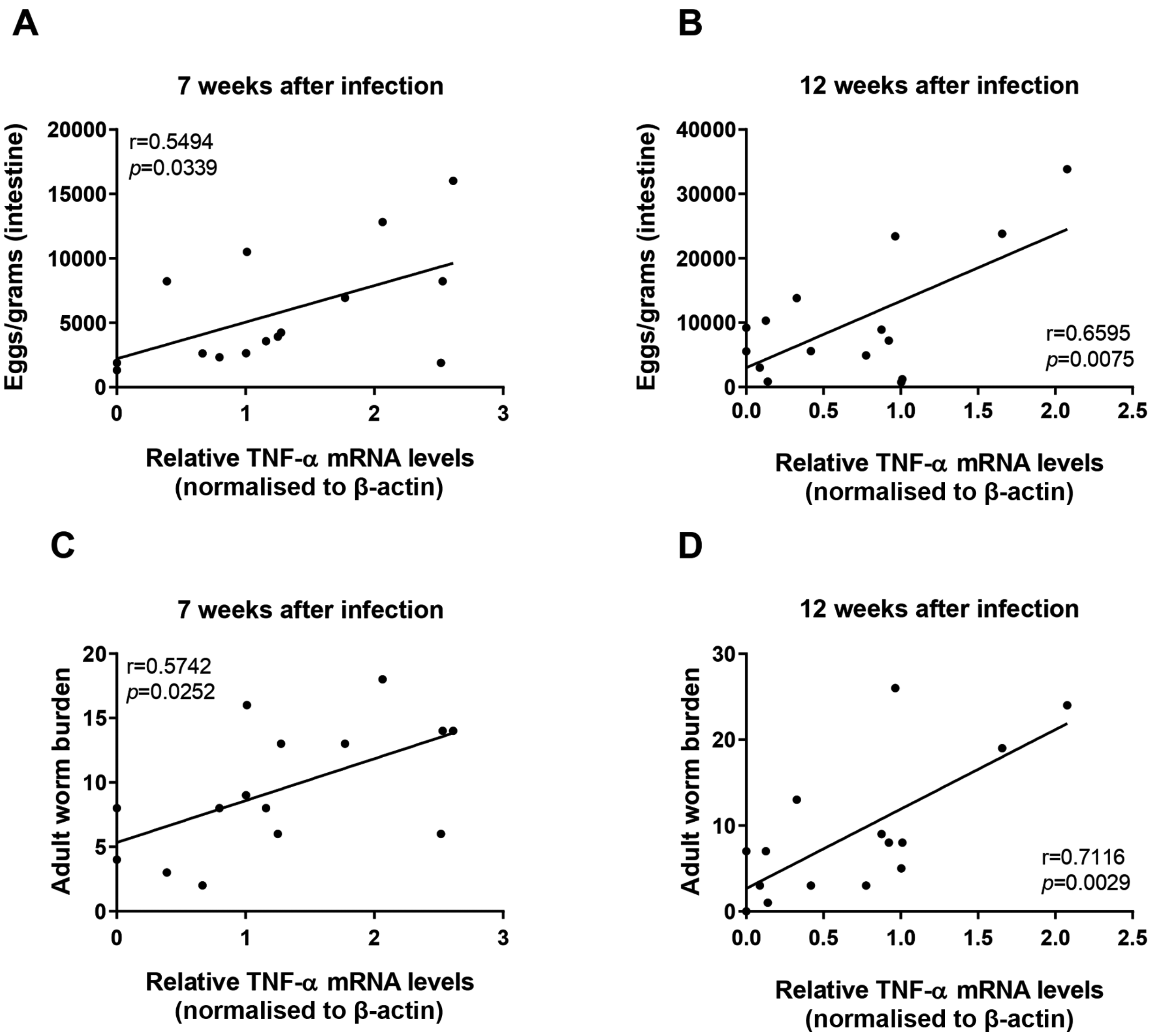

Fig. 4: TNF-α expression is positively correlated with the parasitism and intestine eggs burden. The mRNA expression levels of TNF-α in the liver of Balb/c (n = 5), C57BL/6 (n = 5) and Swiss (n = 5) mice subcutaneously infected with *Schistosoma mansoni* were positively correlated with the eggs/grams in the distal ileum of intestine tissue at (A) 7- and (B) 12-weeks post-infection, and with the number of adult worms in the hepatic portal system at (C) 7- and (D) 12-weeks post-infection. The data are representative of two independent experiments.

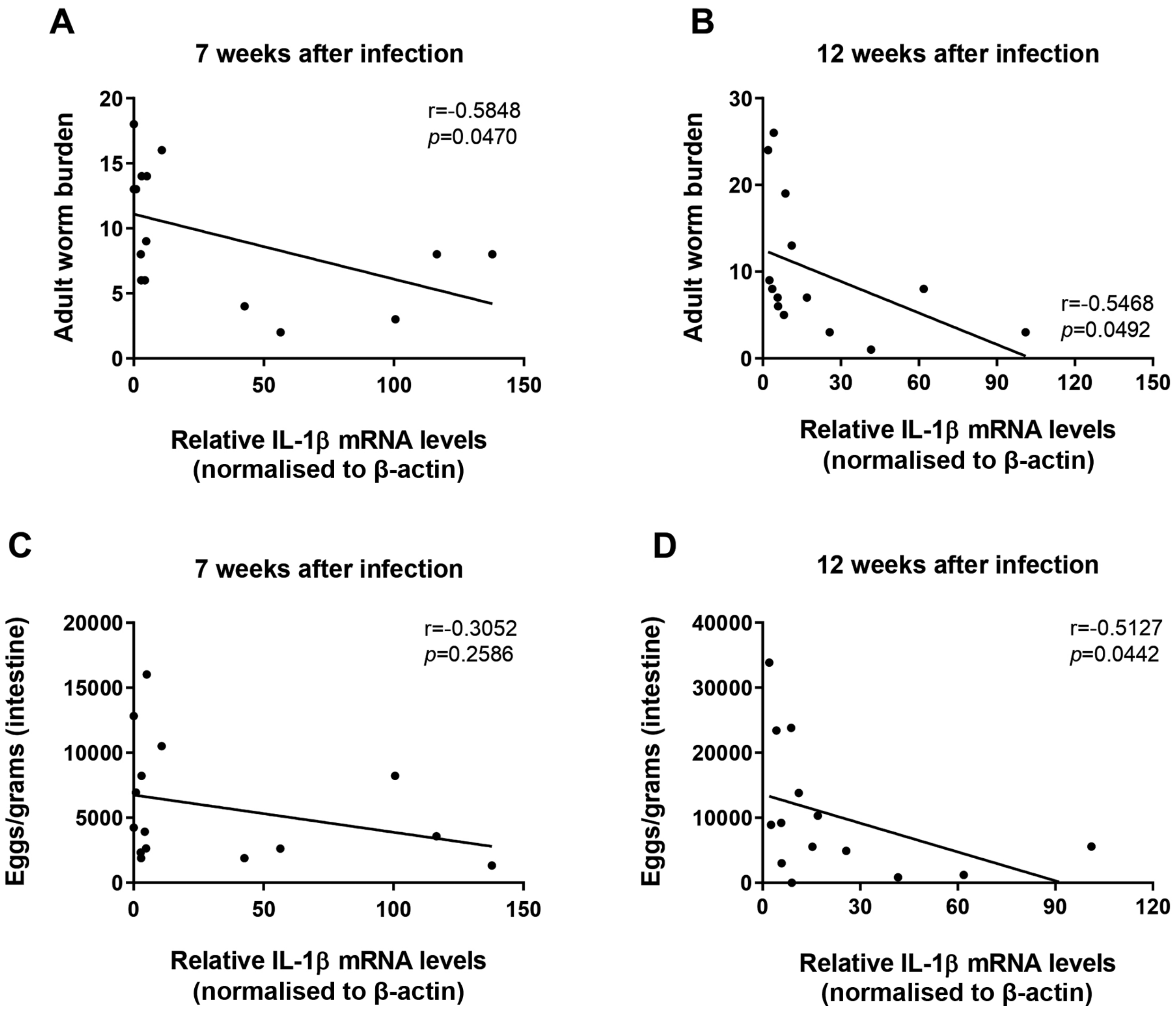

Fig. 5: Pro-IL-1β expression is negatively correlated with parasitism and intestine egg burden. The mRNA expression levels of pro-IL-1β in the liver of Balb/c (n = 5), C57BL/6 (n = 5) and Swiss (n = 5) mice subcutaneously infected with *Schistosoma mansoni* were negatively correlated with the number of adult worms in the hepatic portal system at (A) 7- and (B) 12-weeks post-infection. Correlation analysis of the pro-IL-1β mRNA expression level with the eggs/gram present in the distal ileum of intestine tissue at (C) 7- and (D) 12-weeks post-infection. The data represent two independent experiments.

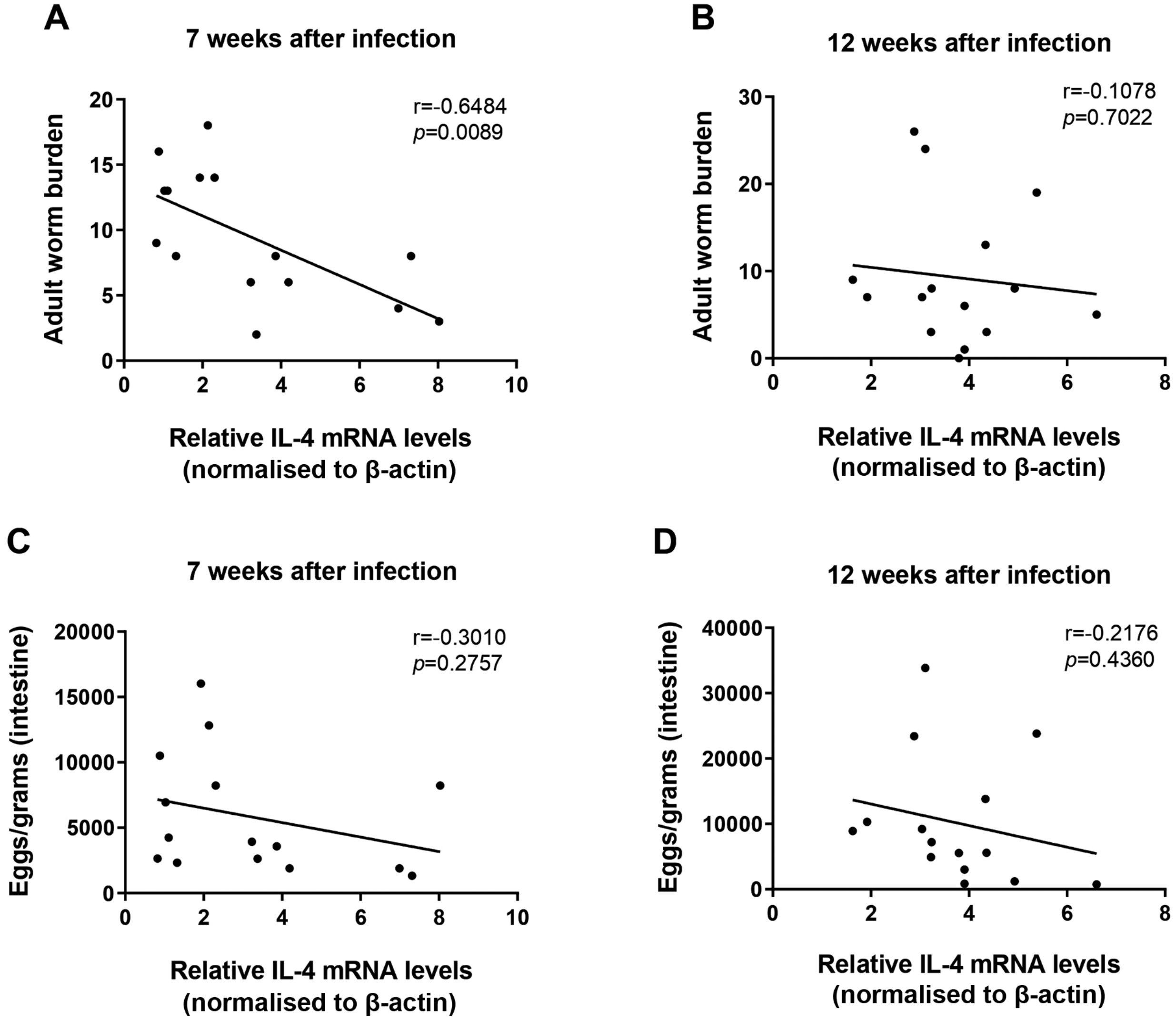

Fig. 6: IL-4 expression is negatively correlated with the parasite burden in the hepatic portal system. The mRNA expression levels of IL-4 in the liver of Balb/c (n = 5), C57BL/6 (n = 5) and Swiss (n = 5) mice subcutaneously infected with *Schistosoma mansoni* were negatively correlated with the number of adult worms in the hepatic portal system at (A) 7-weeks post-infection. (B) Correlation analysis of the IL-4 mRNA expression level with the number of adult worms in the hepatic portal system at 12-weeks post-infection. Correlation analysis of the IL-4 mRNA expression level with the eggs/grams in the distal ileum of intestine tissue at (C) 7- and (D) 12-weeks post-infection. The data are representative of two independent experiments.
